# Hydroxychloroquine Retinopathy in Systemic Lupus Erythematosus: Risk Factors, Screening, and Emerging Biomarkers

**DOI:** 10.1155/joph/8124348

**Published:** 2026-07-13

**Authors:** Yehya Tlaiss, Ali Harajli, Ehab Al Mashtoub, Atef Salame Nasreddine, Tony Badawi, Alaa Tarchichi, Mohamad Tlais

**Affiliations:** ^1^ Department of Ophthalmology, University of Balamand, Beirut, Lebanon, balamand.edu.lb; ^2^ Faculty of Medicine, Lebanese American University, Beirut, Lebanon, lau.edu.lb; ^3^ Faculty of Medicine, University of Balamand, Beirut, Lebanon, balamand.edu.lb; ^4^ Faculty of Medicine, Beirut Arab University, Beirut, Lebanon, bau.edu.lb

**Keywords:** hydroxychloroquine, optical coherence tomography, retinal toxicity, screening strategies, systemic lupus erythematosus

## Abstract

Hydroxychloroquine (HCQ) is a cornerstone therapy for systemic lupus erythematosus (SLE) because of its immunomodulatory and anti‐inflammatory benefits. However, long‐term exposure can cause HCQ‐induced retinopathy, a potentially irreversible adverse effect in which early detection is critical. This review summarizes the literature on the reported prevalence of HCQ‐related retinal toxicity in SLE, highlights consistently supported risk modifiers (including higher daily dose relative to real body weight, longer duration/cumulative exposure, and renal impairment), and synthesizes current screening strategies centered on multimodal structural and functional testing. Spectral‐domain optical coherence tomography (SD‐OCT), fundus autofluorescence (FAF), standard automated perimetry, and multifocal electroretinography (mfERG) are frequently emphasized as complementary tools for earlier detection, while emerging approaches such as OCT angiography‐derived metrics and pharmacokinetic measures remain exploratory. Real‐world screening adherence is often suboptimal, underscoring the need for implementation strategies that align dosing, risk stratification, and ophthalmic monitoring. Future work should standardize diagnostic definitions, clarify optimal screening intervals across risk strata, and prospectively validate promising biomarkers and automated screening workflows.

## 1. Introduction

Systemic lupus erythematosus (SLE) is a chronic autoimmune disease characterized by systemic inflammation and multiorgan involvement, predominantly affecting women of reproductive age [1]. Hydroxychloroquine (HCQ), a 4‐aminoquinoline derivative, remains a cornerstone of SLE management because of its immunomodulatory and anti‐inflammatory effects and its association with reduced disease flares and improved long‐term outcomes [1]. Despite these benefits, long‐term HCQ exposure can be complicated by retinal toxicity, most notably HCQ‐induced retinopathy, which may lead to irreversible vision loss if detected late [1].

HCQ‐induced retinopathy primarily affects the macula, with progressive damage to photoreceptors and the retinal pigment epithelium (RPE). Reported prevalence varies widely across studies, reflecting differences in study design, patient populations, diagnostic definitions, and screening intensity [2]. In long‐term users, cohort data continue to underscore the importance of structured ophthalmic monitoring [2]. Established risk modifiers include higher daily dose (commonly referenced as exceeding 5 mg/kg/day using real body weight), longer duration of therapy, and higher cumulative exposure, as well as impaired renal function; additional systemic factors may influence risk through altered drug clearance and exposure [3].

**FIGURE 1:**
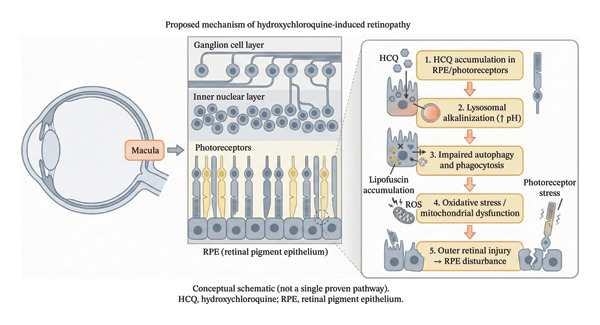
A proposed mechanism linking hydroxychloroquine accumulation in the RPE/photoreceptors to lysosomal alkalinization, impaired autophagy, oxidative stress, and outer retinal injury.

The proposed pathophysiologic mechanism of HCQ‐induced retinopathy is summarized in Figure 1.

Advances in multimodal retinal imaging have improved the ability to detect early toxicity. Optical coherence tomography angiography (OCTA) has been investigated for early microvascular changes (including intereye asymmetry in vessel density and choriocapillaris flow alterations) that could represent potential early biomarkers [4]. Structural imaging, including spectral‐domain optical coherence tomography (SD‐OCT) and swept‐source OCTA (SS‐OCTA), may identify outer retinal and choroidal abnormalities before clinically apparent functional loss in some cohorts, though findings vary by study and methodology [5]. Beyond dose and duration, additional factors have been proposed, including medication interactions and systemic comorbidity, although these findings are not always consistent and may be influenced by differences in population mix and screening protocols [6]. Genetic predisposition has also been explored, including polymorphisms that may influence HCQ metabolism and susceptibility to toxicity, but evidence remains limited and not yet suitable for routine risk stratification [7]. Preexisting retinal disease may complicate screening interpretation and could increase vulnerability in selected patients, as illustrated by case‐based observations [8].

Given the potentially progressive nature of HCQ retinopathy, early detection is critical. Multimodal screening approaches incorporating SD‐OCT, fundus autofluorescence (FAF), multifocal electroretinography (mfERG), and visual field testing are commonly used to improve detection of early changes. However, real‐world adherence to screening recommendations remains suboptimal in multiple settings [9]. In response, the American Academy of Ophthalmology (AAO) revised screening guidance to emphasize risk stratification and the use of complementary structural and functional modalities for earlier detection [10]. Emerging strategies such as pharmacokinetic measures (e.g., blood levels) and automated image‐analysis tools are promising but remain under evaluation and are not yet established as standard of care for ocular toxicity prevention.

Accordingly, this review synthesizes SLE‐focused evidence on (1) reported prevalence of HCQ‐induced retinopathy, (2) consistently supported risk factors versus more exploratory associations, and (3) screening strategies and practical implementation considerations, including what is known about outcomes after dose adjustment or drug discontinuation where data are available.

## 2. Methods

A structured literature review was performed using major biomedical databases (PubMed/MEDLINE, Scopus, and Web of Science) to identify studies relevant to HCQ‐associated retinal toxicity in SLE. Searches used combinations of controlled vocabulary terms and keywords relating to SLE, HCQ, retinopathy/retinal toxicity, and screening modalities (including SD‐OCT, FAF, standard automated perimetry (SAP)/visual fields, mfERG, and OCTA). Reference lists of key articles were also reviewed to identify additional relevant studies.

The focus of this review was on studies reporting SLE‐specific data when available. When mixed rheumatic‐disease cohorts were included in the literature, these findings were interpreted as supportive context and were not assumed to be directly generalizable to SLE‐only populations unless SLE subgroup results were reported separately. Evidence was synthesized narratively due to heterogeneity in study designs, screening definitions, outcome reporting, and risk factor measures across studies.

Because this work summarizes published literature, ethics committee approval was not required.

## 3. Results

### 3.1. Prevalence of HCQ‐Induced Retinopathy in SLE Patients

Reported prevalence of HCQ‐induced retinopathy in SLE varies substantially across the literature, largely due to differences in cohort characteristics (duration of exposure, dosing practices, comorbid risk modifiers), diagnostic criteria, and screening sensitivity. Secondary syntheses and individual observational studies both suggest that clinically detectable toxicity is not rare among long‐term HCQ‐treated SLE patients, but estimates are heterogeneous across settings [11]. For example, Abdelbaky et al. reported a prevalence of 6.3% in a cross‐sectional cohort of 80 SLE patients [12].

In general, larger cohorts using standardized multimodal screening frameworks tend to report prevalence in the low single digits. Petri et al. noted a retinopathy rate of 4.3% in a clinical cohort of 527 SLE patients [13]. Moscarelli et al. reported ocular toxicity in 5.5% of a large monocentric cohort that included SLE and discoid lupus erythematosus (DLE), emphasizing that mixed lupus populations may not be directly comparable to SLE‐only cohorts [2]. Liu et al. reported a definitive retinopathy rate of 4.24% in 259 SLE patients after 5 years of HCQ exposure [14]. In contrast, studies enriched for longer exposure durations have reported higher prevalence; Araujo et al. observed 11.3% prevalence in patients exposed for approximately 15 years, supporting duration as an important modifier [15]. Dabit et al. reported a lower prevalence of 1.73% in a cohort of 634 patients, highlighting how cohort composition and screening definitions can shift observed rates [16].

Additional variability is seen in smaller or specialized populations. Kao et al. observed retinal toxicity in 9.9% of 121 SLE patients on HCQ in a tertiary center cohort [17]. In smaller samples, prevalence estimates have approached the mid‐teens, which may reflect selection factors and/or diagnostic sensitivity [18]. Trefond et al. reported 8.3% retinopathy after 5 years of HCQ use in a 48‐patient case–control study [19]. Overall, published estimates range from approximately 1.73% [16] to values in the mid‐teens in selected cohorts [20], reinforcing the need to interpret prevalence in light of exposure duration, population mix, and screening methodology. Table 1 summarizes representative prevalence studies.

**TABLE 1:** Summary of HCQ‐induced retinopathy prevalence studies.

Study	Year	Sample size (*n*)	HCQ exposure duration	Prevalence of retinopathy (%)	Diagnostic methods used
Cheng et al.	2020	Meta‐analysis	Various	10% overall, 3% severe	OCT, FAF, VF
Abdelbaky et al.	2021	80	≥ 5 years	6.3	SD‐OCT, VF
Petri et al.	2017	527	≥ 5 years	4.3	OCT, FAF, ERG
Moscarelli et al.	2018	504	≥ 5 years	5.5	OCT, FAF, ERG
Araujo et al.	2019	150	≥ 15 years	11.3	SD‐OCT, FAF
Dabit et al.	2020	634	≥ 5 years	1.73	OCT, VF

### 3.2. Risk Factors Associated With HCQ‐Induced Retinopathy

HCQ‐induced retinopathy in SLE appears multifactorial. For clinical clarity, the most consistently supported modifiers relate to dose and cumulative exposure and renal function, while several additional demographic, pharmacokinetic, and immunologic associations remain emerging and require replication across larger cohorts.

**FIGURE 2:**
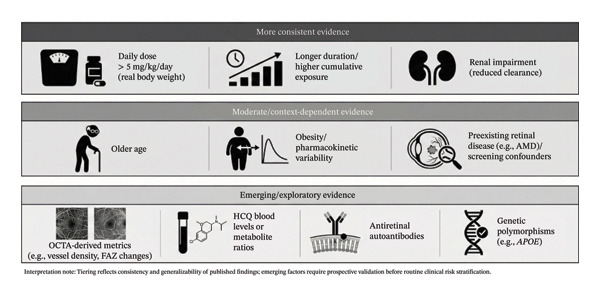
Risk factors for hydroxychloroquine retinopathy in systemic lupus erythematosus (SLE) stratified by strength of evidence.

Figure 2 summarizes reported risk factors for HCQ retinopathy in SLE stratified by strength of evidence, distinguishing consistently supported clinical modifiers from emerging and exploratory biomarkers.

#### 3.2.1. Dose, Cumulative Exposure, and Treatment Duration

Across studies, higher daily dosing relative to real body weight, longer duration of therapy, and higher cumulative exposure are repeatedly associated with increased toxicity risk. Multiple reports support that maintaining the commonly recommended threshold of ≤ 5 mg/kg/day (real body weight) is associated with lower observed rates of toxicity, whereas risk increases substantially with higher dosing and prolonged treatment durations [1]. Consistent with this, cumulative exposure and duration are frequently reported as significant predictors of toxicity [12].

Several studies further link exposure parameters to early structural/vascular signals. Liu et al. observed that total HCQ exposure correlated with changes in OCTA‐derived metrics such as the foveal avascular zone (FAZ), supporting the concept that long‐term exposure is the dominant driver of toxicity risk rather than short‐term fluctuations [1]. In one cohort, risk estimates increased with longer exposure and higher daily doses (> 5 mg/kg) were associated with markedly higher observed toxicity rates compared with dosing at or below the recommended threshold [16]. Kang et al. reported that many patients with retinopathy exceeded a cumulative dose of 1000 g and that long duration of therapy was common among affected patients [21]. Similarly, toxicity has been described at doses modestly above recommended levels, underscoring the importance of precise dose calculation and ongoing adjustment [2].

Even with appropriate daily dosing, long‐term exposure remains relevant. Martin‐Iglesias et al. reported statistically detectable but clinically small retinal thinning over 5 years among 110 SLE patients treated at recommended dosing, supporting relatively short‐ to mid‐term safety under guideline‐consistent regimens [22]. However, guidance cautions that cumulative exposure over longer periods can still lead to toxicity even when dosing recommendations are followed [10]. Some studies suggest that toxicity can occur earlier than 5 years in the presence of additional risk modifiers (e.g., systemic comorbidities), highlighting the need for individualized risk assessment [20]. In real‐world practice, dosing miscalculation remains common; survey data suggest that a substantial proportion of patients receive doses exceeding 5 mg/kg/day when not adjusted to real body weight [23].

#### 3.2.2. Systemic Involvement and Immunological Factors

Disease characteristics may modify risk through cumulative exposure (earlier initiation, longer lifetime therapy) and through systemic complications that affect drug clearance and retinal susceptibility. Some reports suggest higher observed toxicity in SLE subgroups with renal and/or neurologic involvement compared with patients without such complications [14]. Immunologic markers have also been associated with ocular findings in selected cohorts; antiphospholipid‐related markers have been linked to higher observed toxicity rates in some studies, though evidence is not uniform across settings and may reflect subgroup‐specific risk profiles [11]. Antiphospholipid syndrome has likewise been proposed as an additional risk modifier in certain reports [19].

Long‐term exposure remains a central driver, with some studies describing rising observed rates across decades of therapy and associations with vascular/metabolic risk factors, including lupus anticoagulant and hypercholesterolemia, in selected cohorts [15].

#### 3.2.3. Demographic, Anthropometric, and Metabolic Considerations

Age and anthropometric factors may influence risk through pharmacokinetic and comorbidity pathways. Lenfant et al. found that older age, shorter stature, lower creatinine clearance, and reduced hemoglobin levels were associated with increased risk and additionally described geographic ancestry as a potential modifier in their cohort [24]. Other reports describe an age‐related gradient and suggest that higher body mass index may correlate with increased toxicity risk; obesity has been associated with higher HCQ blood levels despite similar dosing, potentially reflecting altered distribution or clearance [25]. These findings suggest that age and body habitus may influence exposure and risk, particularly when dosing is not recalculated to real body weight.

Renal dysfunction is a consistently emphasized modifier. Chronic renal failure has been associated with increased toxicity risk, and lower estimated glomerular filtration rate (eGFR) has been linked to higher HCQ concentrations, potentially compounding risk in older or metabolically compromised patients [26].

#### 3.2.4. Pharmacokinetic Parameters and Biomarkers

Emerging pharmacokinetic data suggest that blood‐based exposure measures and metabolite ratios may identify patients with higher systemic exposure and potentially higher ocular risk, but clinical utility for routine ocular‐toxicity prevention remains under study. Lower blood HCQ/desethylchloroquine (DCQ) ratios and certain medication co‐exposures (including SSRI/SNRI and mycophenolate mofetil) have been discussed as potential modifiers of toxicity severity in selected analyses [19].

Immunologic biomarkers have also been explored. Good et al. reported associations between specific antiretinal autoantibodies and HCQ retinopathy, including a reported increased odds of toxicity in patients with anti‐arrestin antibodies [27]. These findings are promising but should be interpreted as exploratory until replicated in larger prospective cohorts.

#### 3.2.5. Preexisting Ocular Conditions and Real‐World Considerations

Preexisting retinal disease may increase vulnerability and complicate interpretation of screening tests. Case‐based observations suggest that significant retinal comorbidity may be associated with earlier or more pronounced toxicity manifestations [18]. Another report described visual decline in a patient with preexisting age‐related macular degeneration following HCQ exposure, raising concerns about potential exacerbation in susceptible individuals [8]. These observations reinforce the need for careful baseline ophthalmic assessment and individualized screening strategies in patients with ocular comorbidities.

All in all, the literature supports maintaining dosing at ≤ 5 mg/kg/day (real body weight) as a key safeguard, while emphasizing that cumulative exposure, renal function, demographic factors, and selected emerging biomarkers may further refine risk stratification. Table 2 summarizes risk factors reported in the literature.

**TABLE 2:** Risk factors for HCQ‐induced retinopathy.

Risk factor	Details	Impact on retinopathy risk
Dose	> 5 mg/kg/day	4x higher risk of toxicity
Cumulative dose	> 1000 g	Increases risk significantly
Duration of use	> 10 years	3.9% risk at 10 years, 11.3% at 15 years
Renal impairment	Reduced clearance of HCQ	Increased systemic accumulation
Age	> 60 years	Higher risk due to reduced drug metabolism
Genetic factors	APOE polymorphisms	Possible increased susceptibility
Coexisting retinal disease	AMD, CRAO	Exacerbates toxicity
Autoimmune markers	Antiphospholipid syndrome	Higher retinal toxicity rates

### 3.3. Screening, Detection Strategies, and Outcomes

Multimodal screening is repeatedly emphasized for earlier detection of HCQ‐induced retinopathy in SLE, integrating structural imaging with functional assessment to improve sensitivity and reduce missed subclinical disease.

**FIGURE 3:**
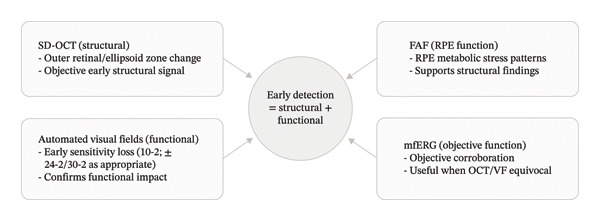
Multimodal screening for hydroxychloroquine retinopathy: what each test contributes.

Figure 3 summarizes the complementary role of structural and functional modalities in multimodal screening for HCQ retinopathy and highlights what each test contributes to early detection.

#### 3.3.1. Innovations in Imaging and Functional Testing

OCTA has been used to explore microvascular changes potentially linked to exposure parameters, including relationships between FAZ metrics and cumulative dosing [21]. FAF may identify early metabolic alterations at the RPE level and has been associated with longer treatment exposure in some reports [12]. Structural imaging (including SD‐OCT) may detect localized outer retinal changes such as photoreceptor layer thinning that can precede symptomatic decline.

Functional testing remains essential, but each modality carries meaningful limitations that must be recognized when interpreting results in clinical practice.

SAP—especially the 10‐2 protocol targeting the central 10 degrees—is the most widely used functional adjunct, and several guidelines endorse it as the preferred perimetric strategy for macular‐focused HCQ monitoring [10]. However, SAP is subject to well‐recognized psychophysical limitations: Test–retest variability can be substantial, patient fatigue degrades reliability over longer test durations, and fixation instability (particularly common in patients with lupus‐related ocular comorbidities) generates artefactual scotomata that may mimic or mask early toxicity. Learning effects in naïve patients further reduce the reliability of a single baseline field, making at least two baseline examinations advisable before attributing subtle paracentral defects to drug toxicity. Moreover, SAP targets cone‐mediated function at photopic luminance levels; very early, predominantly rod‐mediated or parafoveal photoreceptor loss may not be captured until disease is moderately advanced, which partly explains why functional loss on perimetry may lag behind structural changes visible on SD‐OCT. Patients of Asian ancestry represent a clinically important exception to the 10‐2 default. In this population, HCQ toxicity characteristically exhibits a pericentral distribution in which photoreceptor damage extends beyond the central 10 degrees, occurring in a ring outside the classical perifoveal zone rather than the perifoveal pattern predominantly seen in non‐Asian patients [10]. The mechanism underlying this geographic variation is not fully established but may involve differences in macular anatomy, retinal pigmentation, or pharmacokinetic susceptibility. Because the 10‐2 protocol tests only the central 10 degrees, the pericentral damage pattern in Asian patients may be entirely missed without a wider test; accordingly, the 24‐2 program—covering the central 24 degrees with 54 test points and capturing the parafoveal‐to‐pericentral zone—is the recommended perimetric strategy for patients of Asian ancestry [10]. Clinicians should document the patient’s ancestry at baseline and record the appropriate perimetric protocol in the monitoring plan—10‐2 for non‐Asian patients and 24‐2 for Asian patients—since switching protocols mid‐treatment complicates longitudinal comparison and may obscure interval change.

mfERG offers an objective, topographically resolved measure of macular cone function that is independent of patient cooperation and fixation in the conventional perimetric sense. Its principal advantage is the ability to detect subclinical dysfunction—focal reductions in ring‐specific response amplitudes and implicit times—before visual field loss or structural OCT changes become unequivocal [28]. The International Society for Clinical Electrophysiology of Vision (ISCEV) standard for mfERG specifies a 61‐ or 103‐element stimulus array subtending approximately 40–50 degrees of visual angle, recorded with a corneal electrode under photopic conditions (background luminance 100 cd/m^2^, m‐sequence length 2^15^–1), with at least four sweeps per eye and artifact rejection criteria applied to all traces [28]. For HCQ monitoring, the stimulus array is scaled to oversample the central rings, and responses are typically grouped into five concentric rings centered on the fovea. This ring‐based averaging reduces signal noise and allows spatial localization of early macular dysfunction even when the overall waveform amplitude appears preserved.

The mfERG ring ratio is a derived metric that compares the response amplitude (or implicit time) of an inner ring—typically the combined average of Rings 1 and 2, covering the central 7–8 degrees—to that of an outer reference ring, most commonly Ring 5 (approximately 16–22 degrees from fixation). In normal eyes, central cone density and photoreceptor packing generate a relatively larger response per unit area in the innermost rings; a declining ring ratio therefore indicates preferential loss of central macular function relative to the more peripheral retina, consistent with the perifoveal topography of early HCQ toxicity. Jung et al. demonstrated that the Ring 1:Ring 5 implicit time ratio was significantly prolonged in HCQ‐treated SLE patients relative to controls, correlating with cumulative dose and treatment duration, and showed superior sensitivity to early parafoveal dysfunction compared with whole‐field ERG amplitude alone [28]. However, the ring ratio’s diagnostic validity must be understood within its constraints: Normative databases are equipment‐dependent and not universally standardized across ERG systems, amplitude measures are susceptible to media opacities and pupil size variability, and implicit time ratios require meticulous signal‐to‐noise quality control. Interlaboratory comparability therefore remains limited without adherence to ISCEV‐compliant recording parameters, and ring ratio thresholds derived in one equipment/population context should not be uncritically applied in another [28].

It should also be noted that bull’s‐eye maculopathy—the classic funduscopic hallmark of advanced HCQ toxicity characterized by a ring of RPE hypopigmentation surrounding a preserved foveal island—is a late manifestation readily identified on color fundus photography, FAF (as a ring of autofluorescence loss surrounding a hyperautofluorescent center), and SD‐OCT (as a paracentral band of ellipsoid zone and outer nuclear layer thinning). By the time bull’s‐eye maculopathy is clinically apparent, significant and often irreversible photoreceptor loss has already occurred. SD‐OCT is therefore considerably more sensitive for detecting the preclinical outer retinal thinning that precedes the bull’s‐eye stage, and this temporal hierarchy underscores why reliance on funduscopy alone for HCQ screening is insufficient. The earliest structural change detectable on SD‐OCT is typically focal loss of the ellipsoid zone in the perifoveal ring at 2–6 degrees from fixation, which may be present years before a bull’s‐eye pattern becomes ophthalmoscopically visible [10]. The pericentral toxicity distribution in patients of Asian ancestry has direct implications for OCT scanning strategy as well: The standard macular cube protocol (typically covering a 20 × 20‐degree field centered on the fovea) may not adequately sample the pericentral annulus where early damage preferentially occurs in this subpopulation. In Asian patients, supplemental OCT line scans or a widened scan pattern covering the parafoveal‐to‐pericentral region beyond 10 degrees from fixation should be considered, mirroring the rationale for preferring the 24‐2 perimetric protocol in this group [10].

#### 3.3.2. Integration of Blood‐Level Monitoring

In addition to imaging, HCQ blood‐level assessment has been studied as an adjunct to exposure monitoring and risk stratification. In one prospective cohort, higher blood levels correlated with increased toxicity risk even when clinic visit patterns did not correlate directly with toxicity detection [13]. Another study suggested that blood monitoring may help tailor dosing in selected patients rather than relying on clinical examination alone [27]. Overall, blood‐level monitoring is best interpreted as a potential adjunct—particularly in patients with renal impairment or suspected altered clearance—rather than a standalone screening tool.

#### 3.3.3. Impact of Regular Screening on Outcomes

While screening cannot eliminate retinal injury risk, earlier detection can facilitate timely dose adjustment or discontinuation to reduce progression and preserve functional vision. Lee et al. emphasized that screening aims to identify toxicity early rather than fully prevent retinal changes [29]. In practice, earlier detection may support continuation of HCQ where appropriate systemic benefits exist while reducing the likelihood of severe maculopathy through prompt intervention.

Epidemiologic observations suggest that more sensitive modalities increase detection rates. One study reported a higher incidence when sensitive screening tools such as SD‐OCT, 10‐2 visual fields, or FAF were used, reflecting improved detection of early disease rather than necessarily increased biological risk [14]. Additional cohort findings suggest that screening adherence may improve timely identification of ocular involvement [2].

#### 3.3.4. Real‐World Implementation and Screening Frequency

Despite guideline recommendations, real‐world screening adherence is frequently suboptimal. Gubbins et al. reported low screening rates among long‐term HCQ users in a rheumatologic cohort, highlighting system‐level barriers to consistent monitoring [30]. Soroush et al. similarly described ocular toxicity detected in systemic inflammatory disease cohorts, including diagnoses made only after repeated ophthalmologic assessment [31]. These findings support structured referral pathways and risk‐based screening schedules, commonly including baseline evaluation and annual screening after 5 years of therapy (or earlier in higher risk patients), consistent with guideline‐based approaches [10].

#### 3.3.5. Complementing Structural and Functional Assessments

A key theme across studies is the complementary value of structural and functional testing. Structural modalities (e.g., SD‐OCT, FAF, and emerging OCTA markers) provide objective evidence of early retinal change, while functional tests (e.g., 10‐2 visual fields and mfERG) can capture early functional impairment and corroborate imaging findings [10]. Combining modalities helps minimize missed early toxicity and supports more confident clinical decision‐making.

## 4. Discussion

This structured narrative review highlights that HCQ‐induced retinopathy remains a clinically important and partly preventable complication in SLE. Across the literature, reported prevalence varies widely, largely because of heterogeneity in cohort composition (exposure duration, dosing practices, comorbid risk modifiers), diagnostic criteria, and screening intensity. Studies in SLE and mixed lupus/rheumatic cohorts consistently demonstrate that clinically detectable toxicity becomes more frequent with longer exposure and higher risk profiles, although absolute estimates differ by setting and methodology [11–16]. A critical appraisal of the evidence, however, reveals important methodological limitations that qualify the confidence with which findings can be applied to individual patients. The majority of prevalence and risk factor studies are retrospective single‐center or multicenter cohort analyses, which are inherently susceptible to selection bias (tertiary centers screen higher risk patients), ascertainment bias (more intensive screening detects more disease), and survivorship bias (patients who tolerate HCQ longest may be systematically healthier). Case–control studies have provided useful exploratory risk factor data but are limited by small samples, retrospective exposure ascertainment, and referral bias. None of the major screening studies reviewed here were prospectively designed with prespecified diagnostic endpoints or independently adjudicated toxicity definitions, which substantially limits inter‐study comparability. The diagnostic criteria used to define “retinopathy” vary considerably across studies—some require abnormality on a single modality while others mandate multimodal confirmation—inflating apparent cross‐study heterogeneity in prevalence estimates. Furthermore, comparisons of screening modalities across studies are confounded by equipment differences, operator experience, and the absence of standardized reporting thresholds for continuous measures such as OCT layer thickness or mfERG ring ratios. These methodological considerations should inform how clinicians interpret and translate the existing evidence base into practice.

A practical approach to risk reduction begins with accurate dosing and exposure awareness. The AAO recommendations emphasize dosing based on real body weight and identify higher daily dose, long duration, and high cumulative exposure as key risk factors.10 Real‐world dosing and screening adherence remain imperfect in routine practice, and a substantial proportion of patients may exceed recommended dosing thresholds or miss timely screening [23]. These issues likely contribute to delayed detection and more advanced toxicity at diagnosis in some settings.

Screening should remain risk‐based and multimodal. Baseline ophthalmic evaluation prior to or soon after initiating HCQ is helpful to document preexisting macular pathology and establish an anatomic reference [10]. For average‐risk patients, annual screening after several years of continuous therapy is commonly recommended, whereas earlier initiation and/or increased frequency are reasonable in higher risk patients (e.g., higher daily dose, long duration/cumulative exposure, renal impairment, or significant retinal comorbidity) [10]. Structural tests such as SD‐OCT and FAF, combined with functional testing (SAP and/or mfERG), provide complementary information and improve sensitivity for early disease compared with any single modality [10, 28].

Emerging tools should be framed cautiously. OCTA metrics—including microvascular density measures and related parameters—have been explored as potential early signals of toxicity or exposure‐related change, but current evidence remains heterogeneous and largely observational [4, 5]. Pharmacokinetic measures (including HCQ blood levels and metabolite ratios) have been associated with toxicity risk in some cohorts and may be helpful in selected contexts such as suspected nonadherence or altered clearance, but they are not established as a universal routine ocular screening requirement [13, 24–26]. Similarly, exploratory biomarkers such as antiretinal autoantibodies and genetic susceptibility factors have been reported, but require replication and prospective validation before clinical risk stratification can be recommended [19, 27].

Implementation remains a major gap. Multiple reports describe suboptimal screening uptake in real‐world cohorts, indicating that risk‐based screening is not consistently operationalized [30, 31]. Structured referral pathways, reminder systems, and shared‐care protocols can help improve adherence, especially for patients with long‐term exposure or systemic modifiers such as renal impairment [10, 23]. Collaboration should explicitly involve the broader group of clinicians managing SLE (e.g., rheumatologists, internists, nephrologists, and clinical immunologists) alongside ophthalmologists to ensure dosing accuracy, timely referrals, and individualized risk assessment [10, 23, 24].

When retinopathy is suspected or confirmed, decisions about dose reduction or discontinuation must balance ocular risk against the systemic benefits of HCQ for SLE control [29]. Alternative systemic therapies may be clinically appropriate for disease management in selected patients; however, evidence directly demonstrating that switching away from HCQ reduces ocular toxicity risk while preserving equivalent systemic benefit for all patients is limited, and decisions should be individualized with the treating SLE team [29].

**FIGURE 4:**
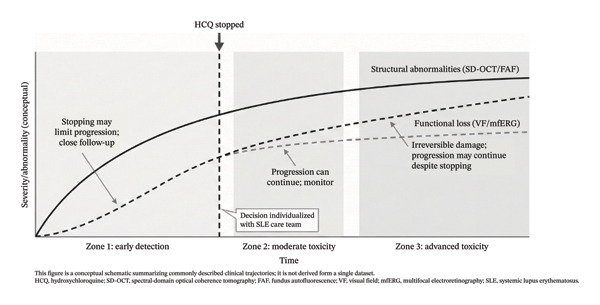
Conceptual course of hydroxychloroquine retinopathy after drug discontinuation.

Figure 4 provides a conceptual schematic of how structural abnormalities and functional loss may evolve after HCQ discontinuation, emphasizing that progression can continue—particularly when toxicity is detected at more advanced stages. This counterintuitive phenomenon deserves explicit discussion, as it is a source of considerable concern for both patients and clinicians who assume that stopping the drug will immediately arrest retinal injury.

The observation that retinal damage may continue or even worsen for months to years after HCQ is stopped is explained primarily by the pharmacokinetic and pharmacodynamic properties of the drug and its principal metabolite, DCQ. HCQ has an extremely long terminal half‐life—estimated at 40–50 days in plasma, but considerably longer in tissue compartments—because it avidly partitions into lysosome‐rich tissues including the RPE and choroid [7]. Following discontinuation, HCQ and DCQ are released slowly from these tissue depots back into the systemic circulation; ocular tissue levels can remain measurable for months to years after the last dose, perpetuating lysosomal alkalinization, impaired autophagy, and ongoing oxidative stress in RPE cells and adjacent photoreceptors. This “pharmacokinetic tail” means that the retina continues to be exposed to cytotoxic drug concentrations even after the patient has stopped taking the medication. Moschos et al. documented progressive outer retinal thinning on SD‐OCT and continued mfERG amplitude decline over a 12‐month follow‐up period after HCQ cessation in patients with established retinopathy, illustrating the clinical relevance of this phenomenon [7]. The degree of postdiscontinuation progression is strongly influenced by the stage of toxicity at the time of detection: Patients diagnosed at an early structural stage (isolated ellipsoid zone irregularity without parafoveal thinning) tend to stabilize relatively quickly after stopping the drug, while those with established parafoveal thinning or bull’s‐eye maculopathy frequently experience measurable further decline. This stage dependence underscores the critical importance of early detection, because prompt discontinuation before widespread photoreceptor loss offers a substantially better prognosis than late intervention.

This review has limitations inherent to the literature base, including heterogeneous definitions of toxicity, variable screening protocols, and inconsistent reporting of effect measures that limit cross‐study comparability [11–16]. Future studies should standardize diagnostic criteria, define exposure metrics consistently, and report comparable outcomes to enable higher confidence synthesis and prospective validation of emerging biomarkers and implementation strategies [4, 5, 19, 27]. A recent comprehensive expert review by Sit and colleagues from the University of Toronto provides a timely update on HCQ ocular toxicity—covering pharmacokinetics, risk stratification, screening modalities, and the diagnosis of retinopathy—and represents a valuable 2026 addition to the evidence base informing the recommendations discussed in this review [32].

## 5. Conclusion

HCQ‐induced retinopathy remains a clinically important complication in SLE that is partly preventable through accurate dosing, risk stratification, and timely screening. The literature most consistently supports higher daily dose relative to real body weight, longer exposure duration and cumulative dose, and renal impairment as key risk modifiers. Multimodal screening that combines structural imaging with complementary functional testing remains central to early detection and clinical decision‐making. Emerging approaches such as OCTA‐derived metrics and pharmacokinetic measures are promising but remain exploratory and require prospective validation before routine adoption. Strengthening real‐world screening adherence and multidisciplinary coordination is essential to preserve vision while maintaining the systemic benefits of HCQ therapy.

## Funding

No funding was received for this manuscript.

## Conflicts of Interest

The authors declare no conflicts of interest.

## Data Availability

The data that support the findings of this study are available from the corresponding author upon reasonable request.
